# Genome-Wide Binding of Posterior HOXA/D Transcription Factors Reveals Subgrouping and Association with CTCF

**DOI:** 10.1371/journal.pgen.1006567

**Published:** 2017-01-19

**Authors:** Ivana Jerković, Daniel M. Ibrahim, Guillaume Andrey, Stefan Haas, Peter Hansen, Catrin Janetzki, Irene González Navarrete, Peter N. Robinson, Jochen Hecht, Stefan Mundlos

**Affiliations:** 1 Max Planck Institute for Molecular Genetics, RG Development & Disease, Berlin, Germany; 2 Berlin-Brandenburg School for Regenerative Therapies, Berlin, Germany; 3 Institute for Medical and Human Genetics, Charité Universitätsmedizin Berlin, Berlin, Germany; 4 Berlin Institute of Health, Berlin, Germany; 5 Department of Computational Molecular Biology, Max-Planck-Institute for Molecular Genetics, Berlin, Germany; 6 Berlin-Brandenburg Center for Regenerative Therapies (BCRT), Charité Universitätsmedizin Berlin, Berlin, Germany; 7 Genomics Unit, Centre for Genomic Regulation, Barcelona, Spain; 8 Universitat Pompeu Fabra (UPF), Barcelona, Spain; The University of Alabama in Huntsville, UNITED STATES

## Abstract

Homeotic genes code for key transcription factors (HOX-TFs) that pattern the animal body plan. During embryonic development, *Hox* genes are expressed in overlapping patterns and function in a partially redundant manner. *In vitro* biochemical screens probing the HOX-TF sequence specificity revealed largely overlapping sequence preferences, indicating that co-factors might modulate the biological function of HOX-TFs. However, due to their overlapping expression pattern, high protein homology, and insufficiently specific antibodies, little is known about their genome-wide binding preferences. In order to overcome this problem, we virally expressed tagged versions of limb-expressed posterior *HOX* genes (*HOXA9-13*, and *HOXD9-13*) in primary chicken mesenchymal limb progenitor cells (micromass). We determined the effect of each HOX-TF on cellular differentiation (chondrogenesis) and gene expression and found that groups of HOX-TFs induce distinct regulatory programs. We used ChIP-seq to determine their individual genome-wide binding profiles and identified between 12,721 and 28,572 binding sites for each of the nine HOX-TFs. Principal Component Analysis (PCA) of binding profiles revealed that the HOX-TFs are clustered in two subgroups (Group 1: HOXA/D9, HOXA/D10, HOXD12, and HOXA13 and Group 2: HOXA/D11 and HOXD13), which are characterized by differences in their sequence specificity and by the presence of cofactor motifs. Specifically, we identified CTCF binding sites in Group 1, indicating that this subgroup of HOX-proteins cooperates with CTCF. We confirmed this interaction by an independent biological assay (Proximity Ligation Assay) and demonstrated that CTCF is a novel HOX cofactor that specifically associates with Group 1 HOX-TFs, pointing towards a possible interplay between HOX-TFs and chromatin architecture.

## Author Summary

*Hox* genes encode transcription factors that determine the vertebrate body plan and pattern structures and organs in the developing embryo. Despite decades of effort and research on *Hox* genes, little is known about the HOX-DNA binding properties *in vivo*. This lack of knowledge is mainly due to the absence of appropriate antibodies to distinguish between different HOX transcription factors. Here, we adapt a cell culture system that allows us to investigate HOX-DNA binding on a genome-wide scale. With this approach, we define and directly compare the genome-wide binding sites of nine posterior HOXA and HOXD transcription factors. We report that the *in vivo* HOX binding specificity differs from the *in vitro* specificity and find that HOX-TFs largely rely on co-factor binding and not only on direct HOX-DNA binding. Finally, we identify a novel HOX co-factor, a genome architecture protein, CTCF suggesting a possible interplay between HOX-TF function and chromatin architecture.

## Introduction

The homeotic genes (*Hox* genes) are key regulators of development. They encode homeodomain transcription factors (HOX-TFs) that are expressed in an overlapping fashion along the anterior-posterior axis in all metazoans [1]. In the vertebrate genome, *Hox* genes are organized in clusters with their order reflecting not only their expression along the anterio-posterior body axis but also their temporal expression onset (spatio-temporal collinearity). In most vertebrates, two rounds of whole-genome duplication have resulted in four clusters of *Hox* genes, coding for a total of 39 HOX-TFs. All HOX-TFs show high levels of sequence conservation between paralog groups (e.g. HOXA9 and HOXD9) and to a lesser extent between genes of the same cluster (e.g. HOXA1 to HOXA13) [reviewed in 2, 3].

In the developing vertebrate limb, the posterior genes of the *HoxA* and *HoxD* clusters (*Hox9-13*) are expressed along the proximo-distal axis following a collinear strategy [4]. Genetic experiments inactivating individual *Hox* genes revealed a remarkable redundancy within paralog groups controlling the development of the proximal (stylopod), middle (zeugopod), and distal (autopod) parts of the limb [5, 6]. For example, neither the homozygous deletion of *Hoxa11* nor *Hoxd11* in mice leads to substantial malformations of the stylo-, or zeugopods. However, deletion of both *Hoxa11* and *Hoxd11* causes a severe truncation of the stylopod and loss of the zeugopod [7, 8]. A similar redundancy is observed between genes of the same cluster. Deletions, in mice, that encompass the entire *Hoxd13* gene cause the adjacent *Hoxd12* to be expressed in a *Hoxd13*-like pattern associated with the functional rescue of the *Hoxd13* deficiency. A similar deletion, removing *Hoxd13* and *Hoxd12* causes *Hoxd11* to be expressed in a *Hoxd13*-like pattern; however, *Hoxd11* is not able to rescue the loss of its two adjacent paralogs [9].

In spite of the insights gained by these elegant series of genetic experiments, the high degree of HOX protein similarity and the overlap of expression domains have hindered the elucidation of the individual HOX-TF functions. HOX-TFs were also included in large biochemical surveys to identify the specific binding sequence of transcription factors [10–12]. Two complementary studies applying protein binding microarrays (PBM) and SELEX-seq on purified DNA-binding domains demonstrated that all posterior HOX-TFs bind to similar AT-rich sequences that vary in their 5’ region but share a characteristic TAAA sequence in their 3’ half. Moreover, two NMR-based studies showed binding of HOXA13 to the HOXD13 site and *vice versa* [13, 14]. Thus, the DNA binding specificity is not sufficient to explain individual HOX-TF function. More recent studies revealed a crucial role for cofactors in HOX-TF specificity. HOX-cofactors were shown to specifically alter the recognition sequence of the HOX-TFs by forming heterodimers [10, 15, 16]. Moreover, the analysis of HOX-cofactor specific binding sites suggested that these altered binding sites might be functionally more relevant for HOX binding than the HOX-TFs binding sites themselves [17]. However, due to high sequence homology, inadequate antibody specificity, and overlapping expression patterns little is known about genomic binding of the different HOX-TFs and how this might relate to their biological function.

Here, we have analyzed and systematically compared the effects of nine limb bud-expressed HOX-TFs (HOXA9-13 and HOXD9-13) on cell differentiation and gene regulation and compare their genome-wide binding characteristics. To mimic the natural HOX environment as closely as possible, we used mesenchymal chicken limb bud cells and moderate retroviral overexpression [18]. In this primary cell culture system (chicken micromass, chMM) the cells normally undergo chondrogenic differentiation; a process that can be altered by virally expressed transgenes [18]. Given the identical cell origin, culture conditions, and antibody use, this system allowed us to assess the distinctive properties of each HOX-TF and compare them to each other.

We find that certain HOXA/HOXD paralog TFs have opposing effects on chondrogenic differentiation and induce distinct regulatory programs in transduced cells. Further, by comparing the genome-wide DNA binding of nine HOX-TFs in this experimental setting, we find that the posterior HOX-TFs can be separated into two groups (Group 1 and Group 2), with distinct binding motifs and distinct associations with cofactors. Finally, we characterized CTCF (the CCCTC-binding factor) as a novel cofactor of HOX-TFs and show that Group 1 but not Group 2 HOX-TFs bind thousands of CTCF-occupied sites in the chicken genome.

## Results

### Posterior HOX-TFs have distinct effects on gene regulation and differentiation of mesenchymal limb bud cells

**Fig 1:**
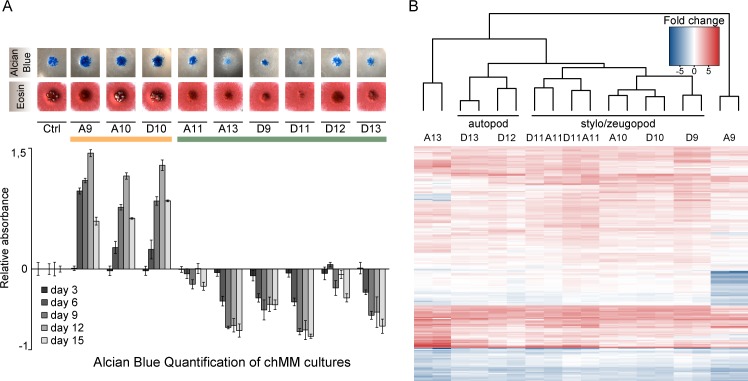
Viral expression of HOX-TFs in chicken micromass culture (chMM) modifies chondrogenic cell differentiation. (A) Individual HOX-TF expressing chMM cultures stained with Alcian blue (top) and Eosin (bottom). Alcian Blue staining of four biological replicates was quantified and compared to mock-infected chMM. Error bars indicate standard deviation from four replicates. (B) Hierarchical clustering of differentially regulated genes in the nine HOX-TF expressing cultures (all RNA-seq shown in replicates). The top 50 differentially regulated genes from each sample were selected (Criteria: p-Val ≤10e-5, base mean≥30, fold change≥2) and for each replicate, the log2-transformed fold changes relative to mock-infected cultures of these 205 genes were subjected to hierarchical clustering.

To systematically compare the function of posterior HOX-TFs, we virally expressed FLAG-tagged versions of each TF in chicken micromass (chMM) cultures. After validating a reproducible, similar, and moderate expression of virally expressed HOX-TFs (S1 Fig), we assessed the effect induced by the different HOX-TFs on chMM cultures. We noticed that some HOX-TFs promoted chondrogenic differentiation (HOXA9, HOXA10, HOXD10), while others inhibited the process (HOXD9, HOXD11, HOXA11, HOXD12, HOXA13, and HOXD13) (Fig 1A).

Interestingly, paralogue HOX-TFs did not always have the same general impact on the chondrogenic differentiation of the chMM. While HOXA9 stimulated chondrogenic differentiation, its paralog HOXD9 inhibited the same process. In contrast, HOXA10 and HOXD10 both promoted chondrogenic differentiation. HOXA11 and HOXD11 both inhibited chondrogenic differentiation, but to a very different extent. Finally, HOXD13 and HOXA13 both strongly inhibited cartilage formation; however, Eosin staining showed that the cell morphology of the HOXA13-expressing chMM was quite distinct from HOXD12 or HOXD13 cultures (Fig 1A).

The simple readout of the chMM morphology showed that the HOX-TFs induce distinct effects on cell differentiation. In order to comprehensively compare the effects on gene expression, we performed RNA-seq of HOX-TF expressing chMM cultures. We used DEseq2 [19] to generate a list of genes that were differentially regulated compared with mock-infected chMM cultures. We then used the genes that were found among the 50 most strongly regulated genes in any of the nine datasets for hierarchical clustering (Fig 1B, S1 Table).

The hierarchical clustering recapitulated some of the main differences found between HOX-TFs that were detected in chMM gross morphology. HOX10 and HOX11 paralogs clustered together, while HOX9 paralogs, which bore striking differences in chMM morphology, clustered apart. Furthermore, the clustering process classified the paralog groups in an order that partially corresponded to their known role in limb development. The clustering separated the stylo-/zeugopod expressed HOX-TFs (HOXD9, HOXA/D10, HOXA/D11) from the autopod expressed HOXD12/13. Two factors, HOXA9 and HOXA13, clustered separately from all other HOX-TFs. This indicates that despite comparable effects on chMM morphology, the regulatory programs induced by HOXA9 and HOXA13 are distinct from the other posterior HOX-TFs. Moreover, the HOX11 paralogs induced transcriptional programs so similar to one another that the clustering algorithm was not able to separate the two replicate datasets from each factor. Interestingly, two genes coding for subunits of the AP1 class of transcription factors, *JUN* and *FOS*, were among the most strongly upregulated genes in all of the datasets, suggesting that they might be direct targets of HOX-TFs.

Finally, we assessed whether the expression of any HOX-TF had a regulatory effect on the HOXA/D gene cluster, since the genetic experiments had suggested the possibility of *HOX* cross-regulation. In fact, we find that all posterior HOX-TFs, with the exception of HOXA9, where able to induce *HOXA/D13* expression at least two fold in comparison to mock-infected chMM cultures. In contrast, HOXA9 had generally a repressive effect on the other *HOX* genes, especially toward its genomic neighbours in cis, *HOXA10* and *HOXA11* (S2 Fig).

Taken together, our analysis shows that, despite high homology and functional redundancy *in vivo*, the direct effects of paralog HOX-TFs in chMM cultures are distinct. While some can be similar (HOXA10/D10 and HOXA11/D11) others can have opposing effects (HOXA13/HOXD13 and HOXA9/D9).

### Genome-wide binding reveals two distinct groups of HOX-TFs

**Fig 2:**
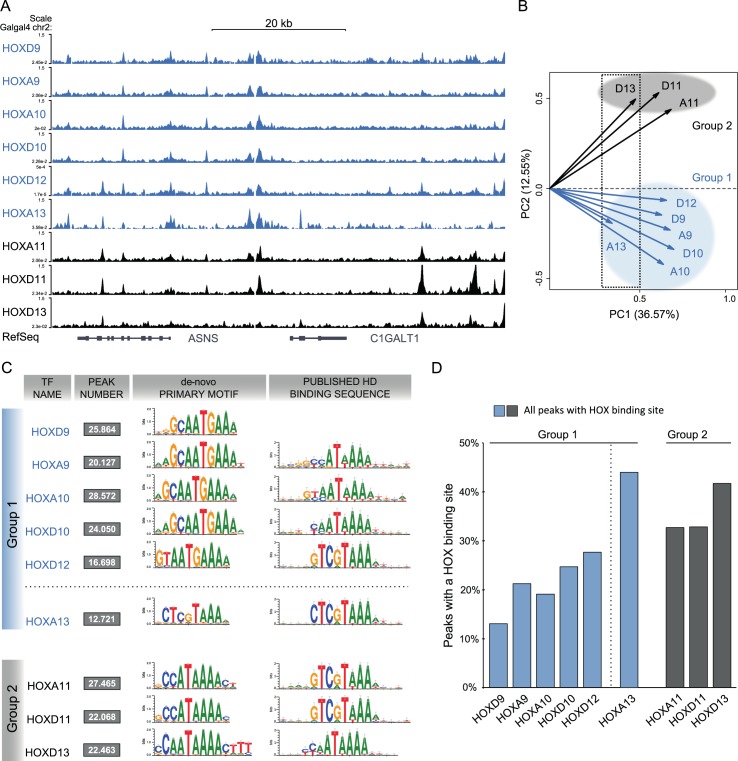
Genome-wide binding profiles of posterior HOX-TFs reveals two groups of binding. (A) ChIP-seq profiles of nine posterior HOX-TFs (Group 1 –blue, Group 2 –black). (B) Principal Component Analysis (PCA) analysis of HOX-TF peaks. HOX13 paralogs cluster separate on PC1 (dotted rectangle). PC2 reveals two distinct groups of HOX-TFs, Group 1 (blue) and Group 2 (black). (C) *De novo* motif analysis for the HOX-TFs. Primary motifs obtained from the top 5,000 peaks in comparison to the previously identified motifs for their respective homeodomains (Berger et al., 2008). Group 1 sequence preferences (except HOXA13, dashed line) are distinct from Group 2. See S1B Fig for additional HOX-like motifs identified in the Top 5,000 peaks. (D) Quantification of peaks carrying binding sites (sequences matching any of the top 3 HOX-TF motifs; FIMO p value≤ 0.0001). Each peak carrying a sequence match is counted only once. Binding site count in top 1,000 and top 10,000 peaks are shown in S1C Fig.

We next wanted to assess whether analogous differences could be observed between paralog groups in their genome-wide binding preferences. We generated ChIP-seq profiles from two biological replicates of virally expressed HOX-TFs in chMM cultures using the αFLAG antibody. We identified between 12,721 and 28,572 binding sites for each of the nine HOX-TFs (Fig 2, S1E and S1F Fig). We first assessed the binding sites shared between HOX-TFs from the same paralog groups by taking the 10,000 strongest peaks for each factor and calculated the pairwise overlap between all HOX-TFs. Similar to the results of the expression analysis, the HOX10 and HOX11 paralogs shared more peaks (78–81% and 85–86%, respectively) than the HOX9 and HOX13 paralogs (65–60% and 29–19%) (S4A Fig).

We then mapped location of HOX-TF binding sites relative to genes. The genomic binding of HOX-TFs respective to genes was similar for all nine factors. The majority of binding sites were either intronic (17–33%) or intergenic (53–60%) and only 9–21% of the peaks were located in annotated promoters (-5kb to +2kb of known TSS).

Next, we performed a principal components analysis (PCA) to compare the datasets in an unbiased way, using the identified peaks as input (Fig 2B). PCA showed that the binding of HOX-TF paralogs seemed to be more similar than their effects on chMM differentiation and gene expression. HOXA13 and HOXD13 were a notable exception as they clustered separately from the other HOX-TFs along PC1 (Fig 2B, dashed box). In addition, they were also very different from one another in PC2. A comparison of all tested HOX-TFs in PC2 revealed a surprising separation into two groups, which neither reflected the effects on cell differentiation and gene expression, nor the sequence homology of the TFs. Group 1 comprised HOXA/D9, HOXA/D10, HOXD12, and HOXA13 (Fig 2B, blue) and Group 2 comprised HOXA/D11 and HOXD13 (Fig 2B, black).

To find a possible cause for this separation, we first tested whether the grouping could be attributed to the sequence-specificity of the TFs. For this we performed *de novo* motif analysis using the peak-motifs algorithm [20] with the 5,000 strongest peaks as input and compared it to the published results from PBM and SELEX-seq (Fig 2C and S4B Fig). This comparison showed a general similarity between *in vitro* and ChIP-seq derived motifs. However, several sequence features had not been detected in the previously published datasets. We found a prominent G at the 5’ end of all Group 1 motifs (HOXA/D9, HOXA/D10, HOXD12, and HOXA13), which had also been detected using SELEX-seq [12]. More striking, we found that the TAAA 3’ end, which is a characteristic of posterior HOX-TFs, changed to a TGAAA in all Group 1 HOX-TF motifs, with the notable exception of HOXA13.

The motifs identified for HOXA13 and HOXD13 were identical to the ones detected in PBM/SELEX-seq. In contrast, the primary motif of HOXA11 and HOXD11 did not overlap with those detected in the corresponding *in vitro* datasets. Specifically, the CCATAAA motif (HOXA/D11) we observed was highly similar to a change in sequence specificity that HOXA10 undergoes when co-binding with PBX4 [10]. Generally, motif analysis for the HOX-TFs identified not only primary motifs but also several alternative HOX-like motifs, suggesting that the DNA-dependent binding of HOX-TFs might be less sequence-driven than other TFs (S4B Fig).

Group 1 and Group 2 HOX-TFs also revealed differences, when we considered the fraction of ChIP-seq peaks that contained a HOX-TFs binding site (Fig 2D and S4C Fig). The number of peaks carrying a HOX-binding site (i.e. matching one of the top three HOX motifs) was relatively low in general, ranging from as little as 15% (HOXD9) to 44% (HOXA13). Interestingly, the three Group 2 HOX-TFs had all high numbers of HOX binding sites in contrast to the Group 1 HOX-TFs, which displayed the lowest number of peaks carrying HOX-TF binding sites. As in the PCA and *de novo* motif analyses, HOXA13 was a notable exception of the Group 1 HOX-TFs, as it contained a high number of peaks carrying binding sites. To exclude the effect of weak and maybe indirect binding sites from the analysis, we performed the same analysis for the 10,000 and 1,000 strongest peaks (S4C Fig). Although the fraction of binding site-containing peaks slightly increased, the general distribution stayed the same.

### *De novo* motif analysis finds putative HOX-cofactors

**Fig 3:**
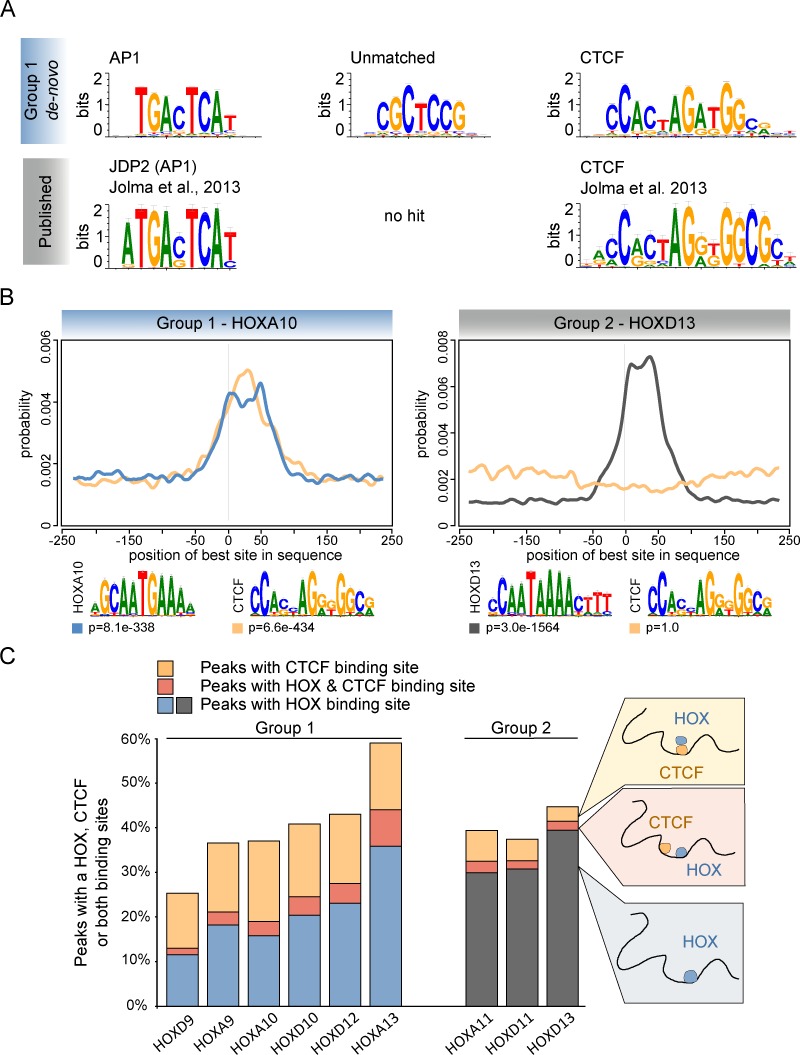
AP1 and CTCF binding sites are overrepresented in Group 1 HOX-TF binding sites. (A) *De novo* motif analysis of all Group 1 HOX-TF peaks (here, HOXA10 results) identifies overrepresented binding sites. A comparison of these motifs to known AP1 and CTCF motifs is shown below. (B) Centrimo analysis identifies the position of best binding site matches in all peak sequences. Blue and black lines indicate enrichment of the given HOXA10 or HOXD13 motif shown below, respectively. Yellow lines indicate enrichment for CTCF motif shown below. (C) The overlap of peaks containing a HOX (Group 1- blue, Group 2- black) or a CTCF (yellow) binding site. The red overlap indicates peaks containing a HOX and a CTCF binding site.

The relatively low numbers of HOX-TF peaks containing HOX binding sites indicated that other factors might contribute to DNA binding. Sequence analysis of ChIP-seq peaks allows not only for the detection of sequence-specific binding sites but also for the identification of putative cofactors. Therefore, we performed a *de novo* motif analysis using all peaks as input and then compared the non-HOX like motifs to the literature and to large TF motif databases (JASPAR [21], footprint DB [22]) (Fig 3).

In Group 2 of HOX-TFs, we were not able to detect any clear cofactor motif. In contrast, we found three putative cofactor motifs in five out of the six Group 1 HOX-TF peak sets. The first motif was the well-characterized TGANTCA AP1 binding site [23] (Fig 3A). A second motif, CGCTCCC/G was detected with high specificity in the HOXA9 and HOXD9 peaks and with lower specificity (but still among the top 5) in the HOXA10, HOXD10 and HOXD12 peaks (S5A and S5B Fig). This motif was particularly enriched in HOXA13 peaks (S8 Fig). We were not able to find matching or similar motifs in the JASPAR and footprint-DB databases, raising the possibility that it either represented the binding site of an uncharacterized TF or a composite binding site recognized by a dimerized TF complex. As a third motif, we detected a 12bp long GC-rich motif in all Group 1 HOX-TF datasets except HOXA13. This motif perfectly matched the known motif of the CCCTC-binding factor (CTCF), a well described TF involved in gene regulation and genome architecture (Fig 3A and S5 Fig)[24].

The *de novo* discovery of cofactor motifs can be masked by the strong overrepresentation of the primary motif. To exclude this possibility, we performed a reverse search and identified and counted all matches to CTCF (Fig 3, S6 Fig) or AP1 (S7 Fig) binding sites in the nine HOX-TF data sets. For the CTCF binding sites, this reverse search revealed a characteristic difference between Group 1 and Group 2 HOX-TFs. Altogether, 14–23% of all Group 1 HOX-TF peaks, but only 5–9% of Group 2 HOX-TF peaks contained a CTCF binding site (S6A Fig). In contrast, we identified AP1 binding sites in about 3–7% of all peaks of the different HOX-TFs and there seemed to be no distinction between Group 1 and Group 2 HOX-TFs (S7 Fig).

Next, we mapped the position of the CTCF binding sites within the HOX-TF peaks and found that in Group 1, but not Group 2, the CTCF sites were located predominantly near the peak summits (Fig 3B and S6B Fig), suggesting a binding mode in which the HOX-TF binds indirectly via CTCF. This was further supported by a discriminatory motif analysis, which revealed that Group 1 HOX-TF peaks contained either a HOX or a CTCF binding site and that only a minority of HOX-TF peaks contained binding sites for both TFs (Fig 3C).

### Group 1 HOX-TFs and CTCF/cohesin co-bind genome-wide

**Fig 4:**
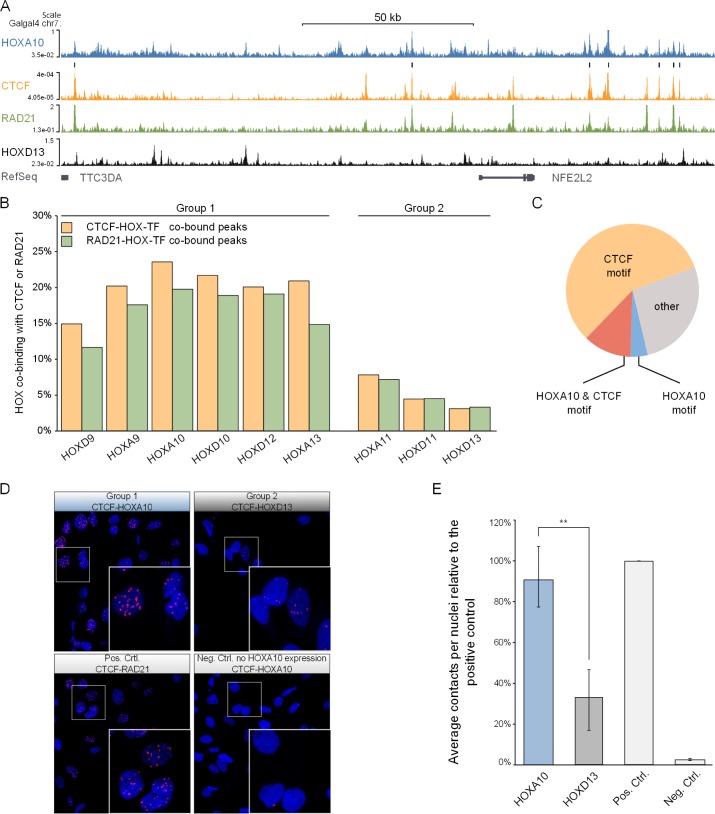
Group 1 HOX-TFs and CTCF/RAD21 share thousands of binding sites throughout the genome. (A) ChIP-seq tracks of HOXA10, CTCF, RAD21, and HOXD13. Black bars above the CTCF-track indicate HOXA10/CTCF co-bound sites. (B) Percentage of HOX-TF peaks overlapping with CTCF (yellow) or RAD21 (green) peaks. (C) The presence of HOXA10 and CTCF binding sites in the HOXA10-CTCF co-bound peaks. (D,E) Proximity Ligation Assay (PLA) in DF1 chicken fibroblasts. (D) Top row: DF1 cells expressing 3xFLAG-HOXA10 (left) or 3 x FLAG-HOXD13 (right). PLA was performed using αFLAG and αCTCF antibodies. Bottom row: Positive Control (left) shows HA-CTCF expressing DF1 cells. PLA was performed with αHA and αRAD21. Negative Control (right) shows non-transfected DF1 cells, PLA performed with αFLAG and αCTCF. (E) Quantification of PLA experiments. Contacts were counted with ImageJ and divided by the number of nuclei in three independent biological replicates (see S11 Fig). The graph shows the percentage of counted contacts relative to the positive control. The standard error of the mean is shown for every sample. A T-test was performed to measure the significance of the contact difference between HOXA10 and HOXD13 (Student’s T p-value<0.005).

Motif analysis indicated that CTCF and Group 1 HOX-TFs might co-bind to many sites throughout the genome. We, therefore, mapped CTCF binding sites genome-wide by virally expressing FLAG-tagged CTCF in chMM cultures (Fig 4) and performed ChIP-seq using the αFLAG antibody. From the same sample, we also performed ChIP-seq for endogenous RAD21, a subunit of the cohesin complex and an important CTCF-cofactor [25]. We identified 22,357 CTCF and 17,589 RAD21 binding sites. Similar to previous reports, CTCF and RAD21 co-bound to 53% of all CTCF and to 67% of all RAD21 peaks. We then tested how many HOX-TF peaks overlapped with CTCF or RAD21 peaks. We observed that the characteristic distinction between Group 1 and Group 2 HOX-TFs could be recapitulated at ChIP-seq binding sites. Indeed, Group 1 HOX-TFs shared between 15% and 24% of their peaks with CTCF (12–20% with RAD21), whereas only 3–8% of Group 2 peaks overlapped with CTCF (3–7% with RAD21) (Fig 4B and S9A, S9B and S9C Fig).

Finally, we set out to find differences between CTCF-HOX shared or HOX-only binding sites using HOXA10 (Group 1) as a representative example. We first tested whether the CTCF-HOXA10 co-bound sites were enriched for promoters or intergenic regions but found no differences between HOXA10-only, HOXA10-CTCF shared or CTCF-only peaks (S10 Fig). We then looked for underlying binding sequences in the 24% of HOXA10 peaks that are shared with CTCF and observed that 69% of them contained a CTCF binding site (23% in all HOXA10 peaks). In contrast, only 16% of the peaks had a HOXA10 binding site (18% in all HOXA10 peaks), suggesting that HOXA10 indirectly binds to these CTCF-shared peaks via CTCF. Taken together, motif analysis of HOX-TF binding sites and ChIP-seq for CTCF/RAD21 both found Group 1, but not Group 2 HOX-TF binding associated with CTCF/cohesin (Fig 4C).

### Group 1 HOX-TFs and CTCF interact in the nucleus

Both, motif analysis and peak overlap strongly suggested an interaction between Group 1 HOX-TFs and CTCF. To test this possibility, we made use of the proximity ligation assay (PLA)[26]. The PLA assay allowed us to assess protein-protein interactions *in situ*, in a quantifiable and sensitive manner. We expressed FLAG-tagged *HOXA10* (Group 1) in chicken DF1 cells and performed the PLA assay using αFLAG antibody and an endogenous αCTCF antibody. We readily detected CTCF-HOXA10 interaction in the nucleus that was almost as strong as the interaction of CTCF with RAD21, which we used as a positive control (Fig 4D). We also performed the same assay with CTCF and the Group 2 HOXD13 protein, for which our ChIP-seq data had predicted a weaker interaction. In this case, we measured a signal above our negative control (DF1 cells expressing CTCF alone), but less than for the CTCF-HOXA10 interaction (Fig 4D and 4E and S11 Fig).

## Discussion

In this study, we systematically compared the effect of nine limb-bud expressed HOX-TFs on the differentiation and gene regulation of primary mesenchymal limb bud cells. Hierarchical clustering of the regulated genes delineated two groups of HOX-TFs: HOX10/11/D9, and HOXD12/HOXD13 that, during limb development, are expressed in the stylo/zeugopod and autopod, respectively. The distinction between these two groups is in accordance with genetic experiments in mice demonstrating that *Hoxd12*, but not *Hoxd11* is able to substitute for a loss of *Hoxd13* [9]. Another interesting observation was that HOXA9 and HOXA13 clustered separately from the other factors. Differences between HOXA/D9 and HOXA/D13 paralogs, in contrast to the more similar HOXA/D10 and HOXA/D11 paralogs, were also apparent in their distinct effects on chMM differentiation. The differences between HOXA9 and HOXD9 (or HOXA13 and HOXD13) might be attributed to the fact that the *HOX9* and *HOX13* paralog groups are the only posterior HOX-TFs which retained all four copies of the genes, thereby reducing the selective pressure on each paralog and allowing their neo-functionalization [3]. The expression analysis further revealed that overexpression of HOXA9 had a negative regulatory effect on most posterior *HOX* genes, especially its direct genomic neighbours in cis, *HOXA10* and *HOXA11*, further confirming the observation from hierarchical clustering that this factor is different from the other studied *HOX* genes. *HOXA/D13* were not negatively regulated by HOXA9 but upregulated by all other HOX-TFs, demonstrating some degree of auto-regulation.

Finally, although *Hoxa13* and *Hoxd13* are partially redundant in loss-of-function experiments [27], we here find that they induce rather different regulatory programs and bind to different genomic regions despite having very similar biochemical DNA binding affinities. This highlights that their divergent molecular function could essentially result in similar developmental outcomes and their observed genetic redundancy reflects an additive effect of both TFs rather than true molecular redundancy.

Our systematic comparison focused on the effects of individual HOX-TFs and their genome-wide binding. However, HOX-TFs are rarely expressed alone *in vivo*, but are rather co-expressed in overlapping patterns and exert their specific function in this biochemical context. Although HOX-TFs induced distinct effects in our experiments, their combinatorial or antagonistic action *in vivo* might play an important role in the developing embryo. Investigation of the *in vitro* sequence specificity of individual HOX-TFs showed that their homeodomains bind largely similar sequences [11, 12]. Subsequent studies, however, revealed that the binding of cofactors changes the original HOX binding site resulting in recognition sites that are markedly different [10, 16, 17]. Both observations are reflected in the results of our ChIP-seq experiments. The low number of direct binding sites in HOX-TF peaks found in our experiments is in concordance with results from *Drosophila*, where low-affinity binding sites for the HOX-TF Ultrabithorax (Ubx) in complex with its cofactor Extradenticle (Exd) were shown to be biologically more significant [17]. Our analysis also highlights the role cofactors play in directing HOX-TF binding. The primary motif for both HOX11 paralogs was in many ways different from the *in vitro* determined monomer specificity and rather revealed a composite binding site like the one bound by a HOXA10-PBX4 dimer [10]. Furthermore, our data indicate a relationship between HOX-TFs and the AP1 class of TFs. AP1 binding sites were found in 5% of all HOX-TF peaks and *JUN* and *FOS* were also strongly upregulated by all HOX-TFs, suggesting a mechanism of cofactor cross-regulation. To our knowledge, AP1 has not been linked to limb patterning or HOX-TFs. However, these factors are known to be involved in a wide array of developmental and cell differentiation processes [28] and our results suggest AP1 may potentially have a role in mediating *HOX*-driven limb patterning.

PCA analysis separated the HOX-TF binding sites in two subgroups along PC2. We tried to identify the underlying cause for this distinction between HOX-TF binding sites and found co-binding with CTCF to correlate with Group 1 HOX-TF binding. We also describe CTCF as a novel cofactor of Group 1 HOX-TFs. CTCF/cohesin are now well-established factors with important functions in the spatial organization of the genome into topologically associating domains (TADs) [29–31]. Among other functions, CTCF/cohesin have been shown to directly mediate enhancer-promoter contacts [25, 32, 33], and the co-occurrence of HOX-TF and CTCF binding sites therefore might result from the presence of CTCF at HOX-bound enhancers. However, the co-occupancy of CTCF/cohesin and HOX-TFs throughout the genome points to a possible role for this type of developmental TFs beyond enhancer-promoter communication. In contrast to our gain-of-function approach, loss of HOXA/D13 TFs has resulted in a miss-regulation of the *HoxD* locus during limb development, where two adjacent TADs regulate the gene expression in the proximal and distal limb, respectively [34, 35]. Specifically, HOXA/D13 proteins did not regulate individual enhancers, but rather restructured the chromatin architecture of the locus in a way so that contacts with one (the telomeric) TAD were repressed, whereas contacts with the other (centromeric) TAD were promoted [35]. A related observation was recently reported in *Drosophila* for CTCF/Cohesin and Smad-TFs, which are the transcriptional effectors of TGFß/BMP signaling [36]. The Smad-TFs co-localized in a CTCF-dependent manner to CTCF binding sites within TADs and might be involved in sculpting the TAD to enable transcriptional regulation. The observed connection of certain developmental TFs with CTCF/cohesin architectural proteins suggests an important fundamental regulatory role for HOX and other TFs that extends beyond the control of individual gene expression.

## Materials and Methods

### Construction of viral vectors and chicken micromass cultures

*HOX* and *CTCF* coding sequences were amplified from chicken embryonic limb buds cDNA (HH27) and cloned into RCASBP-viruses as previously described [18]. DF1 cells were transfected in a 6 cm dish with 3 μg of each RCASBP(A) plasmid using Polyethylenimine (Polyscience Inc. #24765–2) and NaCl. Cells were expanded and stressed on starvation media whereupon the supernatant was harvested on three consecutive days. The supernatant was then centrifuged to produce the concentrated viral particles of high titer, 10^8^ viral particles/ml or higher. The infection of chMM cultures and the histological assessment were performed as described elsewhere [18]. For the quantification of chondrogenesis, cultures after 3, 6, 9, 12 and 15 days post-infection were fixed and stained with Alcian Blue. After 2 washes with 1 x PBS, the quantification of incorporated Alcian Blue was determined by extraction with 6 M guanidine hydrochloride, followed by photometric measurement at A 595 nm. The expression level of cloned constructs was controlled with Western Blot. Cells were lysed using RIPA buffer and immunoblotted using m-αFLAG M2 1:1,000 (Sigma, F1804) (S1D Fig).

### Chromatin preparation and ChIP-sequencing

Chromatin Immunoprecipitation was performed as described previously [18]. Briefly, chMM cultures were harvested after 6 days of culture by adding digestion solution (0.1% collagenase (Sigma, #C9891) and 0.1% Trypsin in 1x PBS) to obtain a roughly single-cell suspension. Cells were taken up in 10 ml cold chMM (DMEM: HAMF11 with 10% FBS, 10% CS, 1% L-glutamine and 1% Penicillin-Streptomycin) medium and fixed for 10 min on ice with 1% formaldehyde. The extraction of nuclear lysate was performed as described in Lee, Johnstone (37) and chromatin was sonicated with a Diagenode Bioruptor (45 cycles—30-sec pulse, 30-sec pause, HI power). For ChIP, 25–35 μg of chromatin was incubated with 6–8 μg of antibody overnight. The next day blocked magnetic beads were added and incubated overnight, followed by 6 washes with RIPA and one with TE buffer [37]. After elution, the preparation of the library for pulled down DNA was performed as described previously [18].

### RNA-sequencing

Cells from harvested chMM cultures were separated prior to fixation of the ChIP samples and RNA was isolated from these cells using an RNaeasy Qiagen kit. RNA-seq libraries were constructed as described previously [18], by selecting for fragment sizes between 300–500 bp and sequenced single-end 50 bp using Illumina technology.

### Proximity ligation assay (PLA)

DF1 cells were transfected with RCASBP(A)-3x FLAG-HOXA10, RCASBP(A)-3x FLAG-HOXD13, or RCASBP(B)-HA-CTCF, respectively. The cells were cultured for at least 6 days to ensure a high cellular infection rate. Upon confluency cells were transferred to 10 mm cover slips and further incubated for one day. Cells were fixed for 10 min with 4% PFA, blocked with TSA (10% horse serum, 0,5% PerkinElmer blocking reagent [#FP1020] and 0.01% Triton-X-100 in 1x DPBS) and incubated with appropriate primary antibodies (in 10% horse serum in 1x DPBST) overnight at 4°C. Primary antibody combinations were: 1) FLAG-HOX and CTCF interaction: m-αFLAG M2 and rb-αCTCF; and 2) HA-CTCF and RAD21 interaction: m-αHA and rb-αRAD21. Antibody concentration for PLA were tested and used as follows: m-αFLAG M2 1:20,000 (Sigma, F1804), m-αHA.11 1:8,000 (BioLegends, #901501), rb-αCTCF 1:20,000 (ActiveMotif, #61311) and rb-αRAD21 1:1,000 (Abcam, ab992).

After primary antibody incubation, the PLA assay was performed using the Duolink In Situ Fluorescence Kit (Sigma, #DUO92101-1KT) according to manufacturer’s instructions. Protein-protein interactions were analyzed by using confocal imaging on a Zeiss LSM700 and the Axiovert Zen software.

For the quantification of PLA experiments, the contacts in several independent frames were counted using ImageJ and divided by the number of nuclei in the frame. The PLA experiments were performed in at least two independent experiments.

## Bioinformatic Analyses

### ChIP-seq

#### Processing and peak analysis

Quality filtering and read mapping were performed as described previously [18]. Reads were mapped against the g*alGal4* reference genome. Reproducible peaks were identified using the MASC2 [38] peak caller and IDR pipeline [39, 40]. For calculating peak overlaps we used bedtools [41]. Summits were extended +/- 150 bp and two peaks were considered overlapping if the overlap was > = 100 bp. The Principal Components Analysis was performed on detected reproducible peaks as described previously [18].

#### Sequence analysis and motif count

*De novo* motif analysis was performed using the peak-motifs algorithm [20] and the sequences +/- 75 bp surrounding the respective peak summits. For counting individual binding sites in the peaks we extracted the sequences +/- 150 bp surrounding the peak summit. Next, the position weight matrix (PWM) of the top three motifs that described the HOX binding site were used together with the FIMO software [42] to obtain the peaks containing a binding site (p < 0.0001). Following this, all peaks that had a sequence match for any of the three motifs were counted as carrying a binding site. A trimmed version of the CTCF-matrix according to the [24](see Fig 2) was used for counting the occurrences of CTCF binding sites. For Centrimo [43] analysis, we used sequences +/- 250 bp of the peak summit and trimmed versions of the PWMs as seen in the motif logos.

### RNA-seq analysis

RNA-sequencing reads were mapped to the chicken reference genome galGal4 using the STAR mapper [44] (splice junctions were based on RefSeq/ENSEMBL gene annotations; options included: alignIntronMin 20, alignIntronMax 500000, outFilterMultimapNmax 5, outFilterMismatchNmax 10, and—outFilterMismatchNoverLmax 0.1). Read counts for individual genes were generated for a gene list combining the RefSeq (galGal4) and ENSEMBL (release 75) gene annotations.

Log2 Fold changes for differential expression were calculated using DEseq2 [19]. The top 50 regulated genes were filtered according to p-value < 10^−5^, a minimum base mean >30 and a fold change >2. For hierarchical clustering, all genes were included that were among the top 50 regulated genes in at least one of the datasets. The log2-transformed fold changes, as compared with control cultures, were then used as the input for the R heatmap3 hierarchical clustering algorithm.

### Estimation of viral expression levels

The RCAS-virus is transcribed as a polycistronic mRNA that is spliced into three distinct isoforms, all containing the HOX-TF message and only one of which is spliced to code for the virally expressed tagged HOX-TF. To deconvolve the HOX-coding splice isoforms from all viral mRNA, we counted the number of RNAseq reads that could be uniquely associated to each of the three possible isoforms and then calculated the fraction of HOX-specific splice variants (SD-SA2). We then multiplied the HOX-TF RPKM with this ratio to obtain the effective RPKM and compared it to the HOX-RPKMs from HH23 wing buds, where all nine relevant HOX-TFs are at least partially expressed [45].

### Deconvolution of triplicated *HOXA* cluster in Galgal4 annotation

In order to generate a correct estimate of the expression values for the *HOXA* genes we had to manually alter the chicken Galgal4 annotation. In the Galgal4 annotation (but not in Galgal3 or Galgal5) the *HOXA* cluster and its genes are annotated in triplicate. We manually changed the annotation so that every additional copy of the gene would be considered a new gene. We then mapped the RNA-seq samples to the altered annotation and used it only to assess the overexpression levels and the *HOX* autoregulation.

## Supporting Information

S1 Fig Hox overexpression levels and the ChIP-seq quality control. (A) Schematic of the RCASBP(A) virus carrying a 3xFLAG tag and a HOX gene. RCASBP(A) virus produces viral polycistronic mRNA that can be either unspliced or spliced in two different variants. Only the SD-SA2 isoform gives rise to the mRNA that will be translated into HOX protein.
(B) In order to compare the expression levels of viral *HOX* in chMM to the endogenous limb bud *HOX* expressions levels, posterior-distal chicken wing buds at the HH23 were dissected as indicated in the drawing. According to Nelson et al. all tested *HOXA* and *HOXD* genes are expressed the dissected tissue in at least ~20% of the cells (*HOXA13*) or in almost all cells (*HOXD11*) (Nelson et al. 1996).
(C) Estimated degree of overexpression by RCASBP(A). Top panel: The fraction of viral mRNA giving rise to HOX protein as calculated from RNA-seq data (see Methods). Middle panel: predicted RPKM values for *HOX* in chMM overexpression experiments on top and RPKM values from distal-posterior wing bud RNA-seq data on bottom.Bottom panel: the fold change of RPKM between posterior-distal wing bud at HH23 and individual chMM experiments for each replicate and averaged between the replicates. Note that HH23 limb buds are a cell population where not all cells express any given *HOX* gene, whereas in chMM every cell expresses the *HOX* gene of interest. *HOXD9* and *HOXA13* are the two lowest expressed factors in our wing bud derived RNA-seq data. *HOXD9* expression is uniform but low at HH23 and *HOXA13* is only expressed in a fraction of all cells (Nelson et al. 1996).
(D) Western Blot (αFLAG) of chMM cultures at day 6 shows uniform expression levels of all nine HOX-TFs. Even though the correct CDS region of *HOXD9* was cloned in to the RCASBP(A) it produces the protein that gives two distinct and reproducible bands. Therefore, we conclude that this represents the HOXD9 protein and a post-translationally modified version rather than the degradation product.
(E) Reproducibility analysis (Irreproducible Discovery Rate) for all ChIP-seq experiments for the HOX-TFs. Self-Consistency Analysis (IDR > 0.01%) for the biological replicates. Bottom: Reproducibility between biological replicates or between “pseudo-replicates” of the pooled replicates. Bold numbers represent the number of reproducible binding sites for each HOX-TF.
(F) log10 IDR plots for the two biological replicates of the nine HOX-TF ChIP-seq experiments. The IDR analysis has been performed according to ENCODE ChIP-seq guidelines (Landt et al. 2012).
(EPS)

S2 Fig Hox autoregulation. (A) *HOXA9-13* and *HOXD9-13* expression was examined in the chMM control and each of the chMM overexpressing cultures. The numbers in the matrix represent expression fold change between the chMM experiments in comparison to the control. Red colour represents upregulation and blue downregulation.
(B) A schematic of *HOX* autoregulation derived from data displayed in A). Only the expression fold change of at least 0.9 fold are plotted in the scheme. Dark lines represent an up- or down regulation of at least one fold and the light lines represent the expression fold change between 0.9 fold to 1 fold.
(EPS)

S3 Fig Enrichment of HOX peaks at genomic locations. Promoters are annotated as 5kb upstream and 2kb downstream of an annotated TSS. Intergenic regions are annotated as all regions that are outside promoter, exon and intron regions. The peaks were examined in a binary fashion and the affiliation to a specific genomic location was determined and plotted.
(EPS)

S4 Fig Pairwise peak overlap and motif analyses for nine posterior ChIP-seq experiments. (A) Pairwise peak overlap of the top 10,000 binding sites for each examined HOX-TF ChIP-seq.
(B) *De novo* motif analysis. Sequences underlying the 5,000 most significant peaks of all reproducible peaks (left) were used as input for the motif analysis. The three most significant motifs from the analysis are shown and in the black boxes (right) the number represents the number of the HOX binding sites detected in the top 1,000 peaks (FIMO, p<10–4) (HOXD9 and HOXA9 have a second motif that is not a primary HOX motif and HOXA10 has third motif that is not a primary HOX motif and have been excluded from the counting). On the left is the comparison to the best fitting HOX motif detected by SELEX-seq (Jolma et al. 2012, (Thomas-Chollier et al. 2012b).
(C) Presence of the binding sites from (B) were counted in the top 1,000, top 10,000 and all reproducible peaks and shown in the bar diagram for Group 1 HOX-TFs (blue) and Group 2 HOX-TFs (black).
(EPS)

S5 Fig *De novo* motif analysis for all reproducible peaks of the nine HOX-TF ChIP-seq dataset. (A,B) Sequences (peak summit +/- 75 bp) underlying all reproducible peaks were used as input for *de novo* motif analysis. The five most significant motifs identified with the Oligos algorithm (top) and Positions algorithm (bottom) with RSAT suite are shown from left to right (Thomas-Chollier et al. 2012b). RSAT parameters were used as default, oligo length = 7. Group 1 HOX-TFs results are shown in (A) and Group 2 HOX-TFs results are shown in (B).
(EPS)

S6 Fig Analysis of CTCF binding sites in HOX-TF peaks. (A) Binding sites matching the published CTCF motif (Barski et al. 2007) were counted in the HOX top 1,000, top 10,000 and all peaks (FIMO, p<10–4). Peaks carrying one or more CTCF binding site were counted only once.
(B) CTCF-like motifs discovered in *de novo* motif analysis, published CTCF-motif, and primary HOX motif are shown left; top to bottom. Centrimo analysis of *de novo* HOX motif (as seen left of the graph) and the published CTCF motif for each HOX ChIP-seq dataset (HOX–blue or black for Group 1 and Group 2, respectively; CTCF–yellow).
(EPS)

S7 Fig Analysis of AP1 binding sites in HOX-TF peaks. (A) Binding sites matching the published JDP2 (AP1) motif (Jolma et al. 2013) were counted in the HOX top 1,000, top 10,000 and all peaks (FIMO, p<10–4). Peaks carrying one or more JDP2 binding site were counted only once.
(B) AP1-like motifs discovered in *de novo* motif analysis, published JDP2 (AP1) motif, and primary HOX motif are shown left, top to bottom. Centrimo analysis of *de novo* HOX motif (as seen left of the graph) and the published JDP2 motif for each HOX ChIP-seq dataset (HOX–blue or black for Group 1 and Group 2, respectively; JDP2 –magenta).
(EPS)

S8 Fig Analysis of GC-rich *de novo* motif in HOX-TF peaks. (A) Binding sites matching the *de novo* detected, GC-rich motif were counted in the HOX top 1,000, top 10,000 and all peaks (FIMO, p<10–4). Peaks carrying one or more binding sites were counted only once.
(B) GC-rich motif discovered in *de novo* motif analysis, consensus GC-rich motif from all HOX-TF ChIP-seq, and primary HOX motif are shown left, top to bottom. Centrimo analysis for *de novo* HOX motif (as seen left of the graph) and the consensus GC-rich motif for each HOX ChIP-seq dataset (HOX–blue or black for Group 1 and Group 2, respectively; GC-rich–green).
(EPS)

S9 Fig Overlap of HOX-TFs and CTCF/RAD21 binding sites throughout the genome. (A) Overlap between the top 1,000, top 10,000 and all HOX-TF peaks with all CTCF peaks.
(B) Overlap between the top 1,000, top 10,000 and all HOX-TF peaks with all RAD21 peaks.
(C) Left: Venn diagram depicts individual and shared binding sites between CTCF and RAD21 genome-wide. Right: Venn diagrams depict the overlap between two co-bound fractions, CTCF-HOX and RAD21-HOX in yellow and green, respectively.
(EPS)

S10 Fig Genomic locations of HOXA10, CTCF and shared HOXA10-CTCF peaks. All HOXA10, all CTCF and only HOXA10-CTCF co-bound peaks and their genomic location distribution. Promoter regions encompass 5kb region upstream and 2kb region downstream of the annotated TSS.
(EPS)

S11 Fig Proximity Ligation Assay (PLA) of HOXA10 and HOXD13 with CTCF. (A, B and C) Triplicate PLA experiments were performed probing the protein-protein interaction of 3xFLAG-HOXA10 and CTCF (top left) and 3xFLAG-HOXD13 and CTCF (top right) with αFLAG and αCTCF antibody. Control PLA experiments were done probing: 1) for POSITIVE control (bottom right): the protein-protein interaction of HA-CTCF and RAD21 using m-αHA and rb-αRAD21 antibody and 2) for NEGATIVE control (bottom left): the protein-protein interaction of FLAG-protein and CTCF using m-αFLAG and rb-αCTCF antibody in DF1 cells not expressing any FLAG-tagged protein. On the right: Contacts were counted with ImageJ and calculated to represent average contacts per nuclei. Standard deviation represents variation between different frames (pictures) counted from the same sample. n = number of frames counted.
(EPS)

S1 Table RNA-seq raw read counts for every annotated gene from the HOX overexpressing chMM cultures and a control chMM culture, in replicates. (TXT)
